# Prevalence of abdominal aortic aneurysms and its relation with cardiovascular risk stratification: protocol of the Risk of Cardiovascular diseases and abdominal aortic Aneurysm in Varese (RoCAV) population based study

**DOI:** 10.1186/s12872-016-0420-2

**Published:** 2016-11-29

**Authors:** F. Gianfagna, G. Veronesi, L. Bertù, M. Tozzi, A. Tarallo, M. M. Ferrario, P. Castelli, RoCAV Project Investigators, Patrizio Castelli, Matteo Tozzi, Marco M. Ferrario, Francesco Gianfagna, Giovanni Veronesi, Lorenza Bertù, Lorenzo Mara, Andrea Montonati, Antonino Tarallo, Marco Franchin, Alessandro Angrisano, Marco Tadiello, Luca M. Quarti, Ilaria Tagliabue, Elena Buscarini, Valeria Farioli, Girolamo Sala, Sonia Agrusti, Alessandro Colombo, Stefania Ferraro, Nicola Rivolta, Gabriele Piffaretti, Rossana Borchini, Marco Conti, Ramona C. Maio, Ursula Andreotta, Martina Ruspa, Laura Turetta, Tiziana Abate, Simona Rossi, Mariapia Ghiringhelli, Federica Quadrini, Nadia Facchinetti, Oriana Dashi, Silvia Mombelli, Davide Mazzoleni, Maria P. Martignoni, Gabriele Caravati, Giancarlo De Luca

**Affiliations:** 1 EPIMED Research Center, University of Insubria, via Rossi 9, Varese, 21100 Italy; 2 Department of Epidemiology and Prevention, IRCCS Istituto Neurologico Mediterraneo Neuromed, via Atinense 18, Pozzilli, 86077 Italy; 3 Vascular Surgery, Varese Hospital - ASST dei Sette Laghi, viale L Borri 57, Varese, 2100 Italy; 4 Department of Surgery and Morphological Sciences, University of Insubria, Via Guicciardini 9, Varese, 21100 Italy; 5 Occupational, Preventive Medicine and Toxicology, Varese Hospital - ASST dei Sette Laghi, viale L Borri 57, Varese, 21100 Italy

**Keywords:** Abdominal aortic aneurysm, Cardiovascular risk, Vascular diseases, Population based study, Screening, ABI, PWV, Electrocardiography, Spirometry, Diet

## Abstract

**Background:**

Recent meta-analyses suggested that screening program for abdominal aortic aneurysms (AAA) in 65-year old males is cost-effective at prevalence of about 1%. Since some events occur also in females and among the youngers, screening could be feasible among those at higher risk, such as smokers or individuals with a family history of AAA. The RoCAV (Risk of Cardiovascular diseases and abdominal aortic Aneurysms in Varese) Project is a population-based study aimed to evaluate AAA prevalence in Northern Italy in males over-65 years as well as among females and younger males, and to identify new markers for risk stratification by collecting a large set of CVD risk factors. The aims of the project are: (i) cross-sectional evaluation of AAA prevalence (ii); evaluation of standard CVD risk score as criteria for selecting subgroup at higher risk to be included in a screening program; (iii) identification of new risk markers and risk score algorithm for AAA and CVD risk stratification; (iv) cost-effective evaluation during the follow-up.

**Methods:**

Males aged 50–75 years and females aged 60–75 years, resident in the city of Varese (Lombardy Region), were randomly selected from the civil registry. Among 5198 successfully invited, 3777 subjects accepted to participate and were finally recruited (participation rate 63.8%) from June 2013 to May 2016. Trained operators administered a computerized anamnestic questionnaire, measured anthropometric parameters (BMI, body circumferences, skinfolds), blood pressure, ankle-brachial index, pulse wave velocity and performed abdominal aortic ultrasound scan, ECG and spirometry. All methods were internationally validated. A blood sample was collected and stored in biobank. A follow-up will be carried out through linkage with electronic records.

**Discussion:**

Participation rate and data quality assessment were as expected and will reasonably allow to reach the project aims. The expected impact in public health of the RoCAV project will be the potential implementation of a AAA screening program to the whole region as well as the formulation of new criteria for risk assessment of AAA and CVD.

## Background

Abdominal aortic aneurysm (AAA) is a very common disease in Western Countries, mainly among males older than 65-years (prevalence of 4–7%), although the prevalence is decreasing in the last years [1, 2]. Large AAA are at increasing risk for rupture, with high mortality (approximately 80% fatal), therefore AAA must be early identified, followed-up and then treated with elective repair.

AAA is a disease well-suited to screening according to the WHO criteria [3]. Recent meta-analyses suggested that screening programs for abdominal aortic aneurysm in 65-year old males reduce all cause and AAA-specific mortality and are cost-effective where disease prevalence is 1% or higher. Recent screenings showed a prevalence of 1.1–1.7% in 65-years old individuals. However data from the general population are available only for Northern Europe, UK and Australia [4–11]. Furthermore, some events occur also in females and in youngers than 65 years. Country-specific data showed that in these subgroups the AAA prevalence is low and then there is no consensus on the cost-effectiveness of a screening program. However it could be suggested in smokers or in presence of a family history [12, 13]. Future studies are needed to identify other risk factors and test their utility as risk stratification markers, to improve feasibility of screening programs as well as to target subgroups at higher risk with primary prevention interventions.

AAA is the result of a loss of elastic lamina and smooth muscle cells, which could be due to inflammatory agents and matrix metalloproteases [14]. The main risk factors found associated with AAA are age, sex, smoking, hypertension and family history of AAA, as well as previous cardiovascular diseases (CVD) [15, 16]. For this reason, the use of standard CVD risk score as well as the presence of a previous CVD could be useful to select subgroups at higher risk to screen for AAA. However, part of the phenotypic variability in the population remains unexplained, probably due to other risk factors or to interactions with the genetic and epigenetic background [17, 18]. Population based studies with the availability of biobank samples could be useful to enlarge the focus on further biomarkers. An AAA risk score algorithm could be evaluated as a criteria to select subgroups of patients at higher risk to be included in a screening program.

Few data are available for Southern European populations, both for prevalence and risk factors, and actually there are no national screening programs. The RoCAV (Risk of Cardiovascular diseases and abdominal aortic Aneurysms in Varese) Project is a population-based cohort study, aimed to evaluate AAA prevalence in Northern Italy even among females and males youngers than 65, and to identify new markers for risk stratification. Furthermore, a huge number of risk factors and diseases was collected, mainly for cardiovascular area, which allow to verify the potential of CVD risk score algorithm [19–21] in AAA risk assessment, as well as to identify new risk markers for a broad spectrum of cardiovascular diseases. Some studies on screening programs of cardiovascular diseases suggested their potential implementation in healthcare settings, although effectiveness has yet to be improved [22]. In fact, conditions at higher cardiovascular risk such as hypertension, hyperglycemia, dyslipidemia, atrial fibrillation are often identified late. The specific aims of the project are: (i) cross-sectional evaluation of AAA prevalence in males starting from 50 years old (50–75 years) and in older females (60–75 years) (ii); potential utility of standard CVD risk score to select subgroups at higher risk to be included in a screening program; (iii) identification of new risk markers and algorithms for AAA and CVD disease risk stratification; (iv) cost-effective evaluation during the follow-up.

## Methods

### Epidemiological design and study population

The RoCAV study is a population-based cohort study. Participants were citizens of Varese (79,793 inhabitants in Lombardy Region, Northern Italy), the main city in a district area of 1 million inhabitants. The civil registry was obtained and 6400 subjects were randomly selected among 50–75 years old males and 60–75 years females, at 30 June 2013, through randomization stratified for sex and 5-years classes (800 subjects for each class) using R software. No exclusion criteria was used. The study was approved by the Varese Hospital Ethical Committee.

### Recruitment

The recruitment of participants started in June 2013 and end in May 2016. An invitation was sent to the participant address by regular mail. The letter included the consent form and the related project information for a preliminary view, along with a suggested date for the examination in our hospital department and a phone number to be contacted for confirmation. An overnight fasting was required. Non-responders were newly contacted by a second letter and then by phone, whenever a phone number was available. The address list was updated periodically to reach citizens who have changed address within the city. In case of refusal, the reported reason was recorded, along with previous diagnosis of AAA. No exclusion criteria were used. A letter was sent to all the GPs of the surrounding area to make them conscious of the possibility for recruitment for their patients and to ask their collaboration in improving the patient participation to the project.

Among 6167 subjects invited, 249 did not received the letter (changed address, death during recruitment). Among the residual 5918 invited, 3777 subjects were finally recruited (65.5 ± 6.7 years; participation rate 63.8%; details for sex and age classes are shown in Table 1). Men showed higher participation rate than women (65.3% vs 61.3%), mainly among the elderly. A total of 692 individuals declared their refusal to participate by phone, mainly due to lack of interest (44.8%; Table 2). Among them, nine (1.3%) subjects declared to have already a known AAA. Differences in demographic characteristics between responders and non-responders are shown in Table 3. Men (59.6% among non-responders vs 63.7% among recruited; *p* = 0.002) and married individuals (67.8% vs 78.2%; *p* < 0.0001) were more willing to participate.

**Table 1:** Participation rates for the overall sample and stratified for sex and age classes

	Males			Females			Total
	Participants (N)	Invited (N)^b^	Rate (%)	Participants (N)	Invited (N)^b^	Rate (%)	Rate (%)
50-54^a^	295	503	58.6	-	-	-	58.6
55–59	490	752	65.2	-	-	-	65.2
60–64	465	698	66.6	315	503	62.6	65.0
65–69	525	777	67.6	479	747	64.1	65.9
≥70^a^	629	950	66.2	579	988	58.6	62.3
All	2404	3680	65.3	1373	2238	61.3	63.8

^a^Invitation letters were sent during all the recruitment time, this caused a shift towards older classes

^b^Subjects who did not receive the letter (changed address, death during recruitment; *n =* 249) were excluded

**Table 2:** Reasons given for refusing to participate (*N =* 692)

Reasons	*N*	%
Not interested in the study	310	44.8
Already under follow-up for a disease	192	27.7
Bedridden or in a retirement home	84	12.1
Recently dead	76	11.0
Recently moved out of the city	21	3.0
Not interested due to previous AAA diagnosis	9	1.3
Total	692	100.0

**Table 3:** Differences between participants and non-responders

		Participants	Non-responders	*p*-value^§^
N		3777	2141	-
Age	Mean (SD)	65.5 (6.7)	65.5 (7.0)	0.95
Men	*n* (%)	2404 (63.7)	1276 (59.6)	0.002
Married^a^	*n* (%)	2900 (78.2)	1331 (67.8)	<0.0001

^§^ *t*-test (age) and chi-square (sex and civil status) were used

^**a**^Missing for 248 subjects (4.2%)

### Data collection

In our hospital departments, a trained operator presented the project and asked to sign the informed consents (study participation and blood sample storage for future analyses). Then a team of trained operators took blood pressure measurements and collected a blood sample, followed by collection of anamnestic, clinical and instrumental data. The methods adhered to the standardized procedures and quality standards of the European Health Examination Survey (EHES) [23], the WHO Multinational MONItoring of trends and determinants in CArdiovascular disease (MONICA) Project [24] and the European Prospective Investigation into Cancer and Nutrition (EPIC) study [25]. All procedures were standardized during training sessions.

A barcode was assigned to the participants to reduce error rate in matching with own electronic folder. A software managed the data collection procedures, as well as recruitment, booking and blood sample storage. Test results were sent to participants and their GPs. Follow-up will start soon using electronic medical records.

#### Anamnestic questionnaires

Standardized, computerized anamnestic questionnaires were administered to the participants, regarding: socio-economic status, lifestyles (smoking, physical activity) and occupational, family (AAA, cardiovascular diseases and cancer in first degree relatives), clinical and pharmacological history. A detailed questionnaire on diet habits was also used (EPIC questionnaire [25]), which includes graphic representations of food types and sizes to facilitate the participant choices. This latter questionnaire was self-administered, with aid from operators to deal with difficulties whenever needed.

#### Anthropometric measurements

Body weight and height were measured on a standard beam balance scale with an attached ruler, in subjects wearing only light indoor clothing (no shoes). Body mass index (BMI) was calculated as weight(kg)/(height(m)^2^. Waist and hip circumferences were measured according to the National Institutes of Health, Heart, Lung, and Blood Guidelines [26]. Mid-upper arm muscle circumference was also measured. A panel of skinfolds was collected (biceps, triceps, subscapular, suprailiac, mid-axillary, chest, abdomen and thigh) using a professional caliper (GIMA Spa, Gessate, Italy), with precision 0.2 mm and range 0–40 mm.

#### Blood pressure and heart rate

Trained medical doctors took blood pressure according to standardized protocol [23, 27]. Measurements were made in a quiet room with comfortable temperature with the participants sitting down for at least 5 min. Blood pressure was measured using a standard sphygmomanometer, with larger cuff where arm circumference was >34.0 cm. The peak inflation level was firstly determined and then the first measurements started 30s later. Blood Pressure was then measured three times on the dominant arm waiting 1 min between measurements, deflating the cuff at a rate of 2 mmHg per second. The radial pulse was palpated and the pulse rate was counted for 60 s, between the first and the second blood pressure measurements.

#### Abdominal aortic imaging

A trained vascular surgeon performed an abdominal aortic ultrasound scan. The sonographers obtained the aortic images using the leading edge-to-leading edge method [28]. Anterior-posterior and transverse diameters were measured using a Esaote scanner with a Convex 3,5 MHz transducer (Esaote, Genoa, Italy), at the following sites: the proximal aorta just below the superior mesenteric artery, the juxtarenal tract at level of renal arteries, the proximal infrarenal aorta 2 cm below the renal arteries, the distal infrarenal aorta 1 cm above the bifurcation, and the point of maximal infrarenal aortic diameter if different than the standard site measurements. A 5-s video of the abdominal tract was also registered. Files were stored and a vascular imaging physician checked all images of ≥25 mm diameters plus a 5% random sample. At the end of the recruitment period, hospitalization and death records were searched for the recruited subjects, along with prevalence and incidence data for the overall city population from which the sample was selected.

#### Ankle-brachial index (ABI) and pulse wave velocity (PWV)

Following the ultrasound scan, participants were tested for ABI and PWV after 5 min rest in supine position. An automated oscillometric measurement BOSO-ABI System (Bosch Sohn GmbH U. Co. KG, Jungingen, Germany) was used, that allows simultaneous arm-leg blood pressure measurements at the 4 limbs (twice, consequent) and then the calculation of ABI and PWV [29].

#### Computerized ECG

Trained physicians performed electrocardiograms following standard procedures [30]. Standard 12-lead resting ECG was measured using a Cardioline electrocardiograph, which acquires synchronized 10-s 12-lead ECG and transmit the waveform data to a workstation in real time with a bluetooth USB connection. Disposable electrodes were used. Digital ECGs were stored in standard communication protocol-ECG format. Electrocardiographs were then evaluated by a cardiologist.

#### Spirometry (peak expiratory flow)

Spirometry was performed by a trained physician using the microQuark USB pc-based spirometer (COSMED, Albano Laziale, Italy), while the patient was upright sitting down. Three tests were obtained and the best result was recorded. Forced vital capacity (FVC), forced expiratory volume in 1 s (FEV1), FEV1/FVC ratio, peak expiratory flow (PEF) and forced expiratory flow at 25, 50% and 75% of FVC (FEF%) were collected.

#### Blood sample, laboratory analyses

A blood sample was collected from antecubital vein between the hours of 8.00 and 9.30 a.m. from participants who had fasted overnight. Fresh samples were split and sent to two laboratories, for laboratory analysis and for storage in the biobank. Blood cell count, serum lipids, blood glucose, creatinine, γGT and transaminases levels were analyzed in the centralized Hospital Laboratory on fresh samples, using commercial reagents and automatic analyzers. LDL-cholesterol was calculated using the Friedewald formula.

#### Biobanking

Blood samples in EDTA tubes (6 ml) and in serum-separating tubes (3.5 ml) were marked with barcodes and transferred to the lab within 1 h and 30 min, under chain-of-custody and then processed within two hours. All tubes were centrifuged 3200 rpm for 10 min, to separate the whole blood in serum (3 ml), plasma (3 ml), buffy coat (1 ml) and red blood cells (2 ml), then collected in 18 cryovials and stored in two refrigerators (−80 °C). A specific software was developed to control the entire procedure from assignment of barcodes to allocation of cryovials, to improve and monitor the process quality and comply with requirements for preserving subject privacy. The RoCAV Biobank was approved by the local Ethical Committee. The procedures met the national and regional guidelines, as well as the international standards [31]. All participants enrolled provided a specific written informed consent to giving blood samples for genetic analysis and further biochemical measurements. Considering refusal to biobank consent and difficulty during blood sample and processing, at the end of recruitment 67,102 cryovials for 3,731 patients were available in the biobank.

#### Follow-up

A follow-up will be carried out through record linkage with electronic records during at least the next 10 years. Total and cause-specific mortality, as well as major CVD, thrombotic events and cancer will be searched using the related ICD-10 codes.

### Outcomes definitions

AAA was defined by a diameter of 30 mm or over in juxtrenal, infrarenal or carrefour site. Further definitions including different diameter thresholds and morphology, specific for sex [32] or standardization using other variables such as anthropometric measures [33] will be considered, evaluating their reliability (AAA progression) using follow-up data. The absence of exclusion criteria allows to include previously known AAA cases in the analyses, as well as to report the estimated total prevalence of the disease in the population along with the screen-detected AAA prevalence.

### Statistical analysis

The sample size calculation was drawn on previous literature in population based studies. Estimated prevalence of AAA was 2–7% among males and 1% among females older than 65-years, with decreasing values for youngers (about 1% for male 50–65 years), therefore we expected to recruit 4000 subjects and to find 100 cases of AAA at the end of the recruitment, which allow to report powerful sex-specific 5-year class prevalence. This sample size is instead more powerful for the association analyses of AAA diameters (continuous data) as well as of all other CVD conditions at higher prevalence than AAA.

Data quality assessment according to MONICA procedures [34] is on-going, details available at the Project website [35].

According to the declared aims data analysis plan will be: (i) cross-sectional evaluation of AAA prevalence in the whole sample and in subgroups at high risk (ii); CVD risk score analysis to stratify the sample in subjects at different CVD risk classes to verify the AAA prevalence, using standard risk score for Italian population (10-year risk score CUORE and SCORE, 20-year risk score CAMUNI-MATISS [19–21]) or other risk score algorithms recalibrated; (iii) analysis of the associations between plausibly related risk factors and AAA as well as CVD, in cross-sectional and longitudinal data using multiple regression models, along with the analysis of discrimination, number needed to screen to prevent a rupture event and net benefit in risk stratification; (iv) cost-effective analysis taking into account follow-up data. All analyses will be carried out using the SAS statistical package (version 9.4 for Windows. SAS Institute Inc. Cary, N.C).

## Discussion

Although screening program for AAA in 65-year old males is reported to be cost-effective in Western Countries [1], only few countries initiated such preventive intervention, such as UK [36], Sweden [37] and US [38]. The main reason of the underuse of AAA screening program is the lack of data for cost-effectiveness estimation. One of the main determinants of screening cost-effectiveness, along with country-specific healthcare settings, is the disease prevalence in the general population, which is not known in most nations, especially for females and subjects younger than 65 years. A further determinant of the cost-effectiveness is the availability of tools for selection of subgroups at higher risk, which lies in the knowledge of risk factors associated with the disease. The RoCAV study will then answer to two main questions, regarding prevalence in Northern Italy among all age and sex classes at risk as well as regarding risk factors or risk score which could be used to select subjects to be included in a screening program.

The prevalence threshold for cost-effectiveness of a screening program is considered to be 0.5–1.6% prevalence in 65-year old males from Western Countries [39]. Several studies collected AAA prevalence data from the general population, using a population-based study design or prevention intervention trials. Most data available are from population of 65-years old males, showing a prevalence of 1.1–1.7% [1]. Prevalence in younger people is lower, however few data are available. For this subgroup, a lower cost-effectiveness threshold should be considered, due to higher QALY gained with a screening program. A recent meta-analysis showed that prevalence in females is greater than 1% only after 70 years [40], with a large heterogeneity across countries. It is noteworthy that recently a decline in AAA prevalence was observed, since the improved CVD risk management and a decline in smoking. However, at the same time, surgical techniques improved and longevity increased, contributing to decrease the expected threshold for cost-effectiveness in different context [1]. AAA prevalence is then highly heterogeneous across countries and the epidemiological context are changing. Collecting regional data from the general population is strongly requested. The main aim of the RoCAV was to verify the AAA prevalence for the first time in a Northern Italian population, using a population-based approach and standardized methods for a screening program, for a rapid translation in the real public health context.

Cost-effectiveness of a screening program could be improved tailoring the intervention to a subgroup with a higher prevalence, identified basing on risk factors prevalence. The risk factors associated with AAA risk are smoking, family history of AAA and hypertension, along with age and sex. In different context, elderly males having one of more of this risk factors are suggested to undergo an ultrasound imaging scan of the abdominal aorta. Smoking is the main risk factor, which explain about 3 out of 4 cases. Having a previous CVD is suggested to be an important risk marker [15, 16], due to shared risk factors between AAA and CVD. The association with more CVD risk factors and with CVD history suggests to use the same risk assessment tool used for major CVD, like CVD risk scores. This was the second aim of the RoCAV study, very recently addressed also by Jones et al. in selected populations [41]. Our study will address this question in the context of a large sample, randomly selected from the general population.

However, part of the AAA phenotypic variability in the population remains unexplained, due to the effect of other known risk factors, exerting a small effect, or less known conditions not yet well studied, with a strong effect. The large set of data collected in the RoCAV study, their novelty (PWV, ABI, diet scores), the validated tools used, the possibility to measure new biomarkers in samples stored in biobank (metabolomics and proteomics) will address this question. Furthermore, it is expected than a large impact would have epigenetic and genetic mechanisms [17, 18], alone or in interaction with environmental variables, both measurable in biobank samples. Therefore, the availability of this large set of data will allow to model an AAA risk score algorithm for risk assessment, potentially useful to select subgroups of patients at higher risk to be included in a screening program, as for risk stratification of major CVD.

Despite the importance of AAA, the main burden of disease in public health is represented by CVD and cancer. The large set of CVD-related risk factors allow to study also new markers for CVD, as well as for any other disease present in the general population. Conditions other than AAA, such as atrial fibrillation, could be in fact potentially prevented with a screening program in the general population or in subgroups at high risk [22].

The RoCAV study has several advantages. It is a paperless study, data collection (questionnaires as well as ECG, spirometry, ABI-PWV and ultrasound parameters) was totally computerized, reducing potential errors during a delayed data input. Moreover, the use of barcodes to identify patients limited the possibility of wrong assignment to the patient electronic record. Furthermore, a large set of data were collected, using previously validated methods and innovative tools. Finally, the availability of sample stored in biobank for future analysis will allow the evaluation of OMICs and other biomarkers.

The expected impact of the RoCAV project is: 1) estimation of the AAA prevalence in the general population of Northern Italy in males older than 50 years and in females older than 60 years and the potential implementation of a screening program to the whole region; 2) evaluation of the clinical utility of standard CVD and AAA risk scores in AAA risk stratification, to improve selection of subjects to be included in the AAA screening program; 3) long-term estimation of cost-effectiveness of the screening program in the local context, considering the possibility to reduce also CVD-specific and all-cause mortality.

## Abbreviations

AAA: Abdominal aortic aneurysm
ABI: Ankle brachial index
BMI: Body mass index
EHES: European Health Examination Survey
EPIC: European Prospective Investigation into Cancer and Nutrition
FEF%: Forced expiratory flow at 25, 50% and 75% of FVC
FEV1: Forced expiratory volume in 1 s
FVC: Forced vital capacity
MONICA: Multinational MONItoring of trends and determinants in CArdiovascular disease
PEF: Peak expiratory flow
PWV: Pulse wave velocity
RoCAV: Risk of Cardiovascular diseases and abdominal aortic Aneurysms in Varese
